# Analysis of risk factor domains in psychosis patient health records

**DOI:** 10.1186/s13326-019-0210-8

**Published:** 2019-10-31

**Authors:** Eben Holderness, Nicholas Miller, Philip Cawkwell, Kirsten Bolton, Marie Meteer, James Pustejovsky, Mei-Hua Hall

**Affiliations:** 1 Psychosis Neurobiology Laboratory, McLean Hospital, Harvard Medical School, Mill St, Belmont, MA, USA; 2 Brandeis University Department of Computer Science, South St, Waltham, MA, USA

**Keywords:** Natural language processing, Risk prediction, Machine learning, Electronic health record, Psychotic disorders

## Abstract

**Background:**

Readmission after discharge from a hospital is disruptive and costly, regardless of the reason. However, it can be particularly problematic for psychiatric patients, so predicting which patients may be readmitted is critically important but also very difficult. Clinical narratives in psychiatric electronic health records (EHRs) span a wide range of topics and vocabulary; therefore, a psychiatric readmission prediction model must begin with a robust and interpretable topic extraction component.

**Results:**

We designed and evaluated multiple multilayer perceptron and radial basis function neural networks to predict the sentences in a patient’s EHR that are associated with one or more of seven readmission risk factor domains that we identified. In contrast to our baseline cosine similarity model that is based on the methodologies of prior works, our deep learning approaches achieved considerably better F1 scores (0.83 vs 0.66) while also being more scalable and computationally efficient with large volumes of data. Additionally, we found that integrating clinically relevant multiword expressions during preprocessing improves the accuracy of our models and allows for identifying a wider scope of training data in a semi-supervised setting.

**Conclusion:**

We created a data pipeline for using document vector similarity metrics to perform topic extraction on psychiatric EHR data in service of our long-term goal of creating a readmission risk classifier. We show results for our topic extraction model and identify additional features we will be incorporating in the future.

## Background

Psychotic disorders typically emerge in late adolescence or early adulthood [1, 2] and affect approximately 2.5-4% of the population [3, 4], making them one of the leading causes of disability worldwide [5]. A substantial proportion of psychiatric inpatients are readmitted after discharge [6]. Readmissions are disruptive for patients and families, and are a key driver of rising healthcare costs [7, 8]. Reducing readmission risk is therefore a major unmet need of psychiatric care. Developing clinically implementable machine learning tools to enable accurate assessment of risk factors associated with readmission offers opportunities to inform the selection of treatment interventions and implement appropriate preventive measures.

In psychiatry, traditional strategies to study readmission risk factors rely on clinical observation and manual retrospective chart review [9, 10]. This approach, although benefitting from clinical expertise, does not scale well for large data sets, is effort-intensive, and lacks automation. An efficient, more robust, and cheaper alternative approach based on Natural Language Processing (NLP) has been developed and met with some success in other medical fields [11]. However, this approach has seldom been applied in psychiatry because of the unique characteristics of psychiatric medical record content.

There are several challenges for topic extraction when dealing with clinical narratives in psychiatric EHRs. First, the vocabulary used is highly varied and context-sensitive. A patient may report “feeling ‘really great and excited” – symptoms of mania – without any explicit mention of keywords that differ from everyday vocabulary. Also, many technical terms in clinical narratives are multiword expressions (MWEs) such as ‘obsessive body image’, ‘linear thinking’, ‘short attention span’, or ‘panic attack’. These phrasemes are comprised of words that in isolation do not impart much information in determining relatedness to a given topic but do in the context of the expression.

Second, the narrative structure in psychiatric clinical narratives varies considerably in how the same phenomenon can be described. Hallucinations, for example, could be described as “the patient reports auditory hallucinations,” or “the patient has been hearing voices for several months,” amongst many other possibilities.

Third, phenomena can be directly mentioned without necessarily being relevant to the patient specifically. Psychosis patient discharge summaries, for instance, can include future treatment plans (e.g. “Prevent relapse of a manic or major depressive episode.”, “Prevent recurrence of psychosis.”) containing vocabulary that at the word-level seem strongly correlated with readmission risk. Yet at the sentence-level these do not indicate the presence of a readmission risk factor in the patient and in fact indicate the absence of a risk factor that was formerly present.

Lastly, given the complexity of phenotypic assessment in psychiatric illnesses, patients with psychosis exhibit considerable differences in terms of illness and symptom presentation. The constellation of symptoms leads to various diagnoses and comorbidities that can change over time, including schizophrenia, schizoaffective disorder, bipolar disorder with psychosis, and substance use induced psychosis. Thus, the lexicon of words and phrases used in EHRs differs not only across diagnoses but also across patients and time.

Taken together, these factors make topic extraction a difficult task that cannot be accomplished by keyword search or other simple text-mining techniques.

To identify specific risk factors to focus on, we not only reviewed clinical literature of risk factors associated with readmission [12, 13], but also considered research related to functional remission [14], forensic risk factors [15], and consulted clinicians involved with this project. Seven risk factor domains – Appearance, Mood, Interpersonal, Occupation, Thought Content, Thought Process, and Substance – were chosen because they are clinically relevant, consistent with literature, replicable across data sets, explainable, and implementable in NLP algorithms. These seven risk factor domains collectively cover the essential clinical aspects of a patient’s symptoms and functioning. Although hospitals may differ in terms of narrative structure, all of a patient’s admission notes and discharge summaries typically include text of these seven domains. Many hospitals in the US include each of these risk factors as a heading or subheading.

In our present study, we evaluate multiple approaches to automatically identify which risk factor domains are associated with which sentences in psychotic patient EHRs^1^. We perform this study in support of our long-term goal of creating a readmission risk classifier that can aid clinicians in targeting individual treatment interventions and assessing patient risk of harm (e.g. suicidal risk, homicidal risk). Unlike other contemporary approaches in machine learning, we intend to create a model that is clinically explainable and flexible across training data while maintaining consistent performance.

To incorporate clinical expertise in the identification of risk factor domains, we undertake an annotation project, detailed in the “Annotation task” subsection of the “Methods” section. We identify a test set of over 5000 EHR sentences which a team of three domain-expert clinicians annotate sentence-by-sentence for relevant risk factor domains. The “Inter-Annotator agreement” subsection of the “Methods” section describes the results of this annotation task. We then use the gold standard from the annotation project to assess the performance of multiple neural classification models trained exclusively on institutional EHR data, described in the “Results” section. To further improve the performance of our model, we incorporate domain-relevant MWEs identified using all in-house data.

### Related work

McCoy et al. [16] constructed a corpus of web data based on the Research Domain Criteria (RDoC)[17], and used this corpus to create a vector space document similarity model for topic extraction. They found that the ‘negative valence’ and ‘social’ RDoC domains were associated with readmission. Using web data (in this case data retrieved from the Bing API) to train a similarity model for EHR texts is problematic since it differs from the target data in both structure and content. Based on reconstruction of the procedure, we conclude that many of the informative MWEs critical to understanding the topics of sentences in EHRs are not captured in the web data. Additionally, RDoC is by design a generalized research construct to describe the entire spectrum of mental disorders and does not include domains that are based on observation or causes of symptoms. Important indicators within EHRs of patient health, like appearance or occupation, are not included in the RDoC constructs.

Rumshisky et al. [18] used a corpus of EHRs from patients with a primary diagnosis of major depressive disorder to create a 75-topic Latent Dirichlet Allocation (LDA) topic model that they then used in a readmission prediction classifier pipeline. Like with McCoy et al. [16], the data used to train the LDA model was not ideal as the generalizability of the data was narrow, focusing on only one disorder. Their model achieved readmission prediction performance with an area under the curve of.784 compared to a baseline of.618. To perform clinical validation of the topics derived from the LDA model, they manually evaluated and annotated the topics, identifying the most informative vocabulary for the top ten topics. With their training data, they found the strongest coherence occurred in topics involving substance use, suicidality, and anxiety disorders. But given the unsupervised nature of the LDA clustering algorithm, the topic coherence they observed is not guaranteed across data sets.

## Methods

### Data

Two non-overlapping but highly compatible datasets were used for training (Research Patient Data Registry, RPDR) and for testing (McLean Meditech) of our models. Our test set (McLean) consists of a corpus of discharge summaries, admission notes, individual encounter notes, and other clinical notes from 220 patients in the OnTrack^TM^ program at McLean Hospital. OnTrack^TM^ is an outpatient program, focusing on treating adults ages 18 to 30 who are experiencing their first episodes of psychosis. The length of time in the program varies depending on patient improvement and insurance coverage, with an average of two to three years. The program focuses primarily on early intervention via individual therapy, group therapy, medication evaluation, and medication management. See Table 1 for a demographic breakdown of the 220 patients, for which we have so far extracted approximately 240,000 total EHR sentences spanning from 2011 to 2014 using Meditech, the software employed by McLean for storing and organizing EHR data.

**Table 1:** Demographic breakdown of the target cohort

Mean Age (2014)	20.7
Gender (Male)	79%
Race	
Asian	6%
Black	7%
Caucasian	77%
Latino	5%
Multiracial	5%
Insurance (Public)^2^	5.5%
30-day Inpatient Readmission Rate	14%

These patients are part of a larger research cohort of approximately 1800 psychosis patients, which will allow us to connect the results of this EHR study with other ongoing research studies incorporating genetic, cognitive, neurobiological, and functional outcome data from this cohort.

We also use an independent, non-overlapping data set for identifying training data for our vector space model, comprised of EHR texts queried from the RPDR, a centralized regional data repository of clinical data from all institutions in the Partners HealthCare network (e.g., Massachusetts General Hospital, Brigham and Women’s Hospital). These records are highly comparable in style and vocabulary to the McLean data set. The corpus consists of discharge summaries, encounter notes, and visit notes of patients admitted to the system’s hospitals with psychiatric diagnoses and symptoms, totaling approximately 8,000,000 EHR sentences consisting of 340,000,000 tokens. This breadth of data captures a wide range of clinical narratives, creating a comprehensive foundation for topic extraction.

After using the RPDR query tool to extract EHR sentences from the RPDR database, we created a training corpus by categorizing the extracted sentences according to their risk factor domain using a lexicon of 120 keywords that were identified by the clinicians involved in this project. Certain domains – particularly those involving thoughts and other abstract concepts – are often identifiable by MWEs rather than single words. The same clinicians who identified the keywords manually examined the bigrams and trigrams with the highest Term Frequency – Inverse Document Frequency scores (TF-IDF) for each domain in the categorized sentences, identifying those which are conceptually related to the given domain. We then used this lexicon of 775 keyphrases to identify more relevant training sentences in RPDR and treat them as (non-stemmed) unigrams when generating the matrix (see supplementary data). By converting MWEs such as ‘shortened attention span’, ‘unusual motor activity’, ‘wide-ranging affect’, or ‘linear thinking’ to non-stemmed unigrams, the predictive value of these terms is magnified. In total, we constructed a corpus of roughly 85,000,000 tokens across 2,100,000 EHR sentences for training our model.

#### Annotation task

In order to evaluate our models, we created an annotated test corpus McLean-specific EHR data extracted from Meditech. 5154 sentences were annotated by three licensed clinicians for the clinically relevant domains described in Table 2. The corpus was selected by clinicians (P. C. and K. B.) who treat patients at McLlean OnTrack program and M.H.H who conducts clinical research at the McLean Psychotic Disorders Division. It is comprised entirely of McLean-specific EHR data, which are disjoint from the RPDR but are highly compatible in style and vocabularies with the RPDR dataset.

**Table 2:** Annotation scheme for the domain classification task

Domain	Description	Example	Example Keywords
Appearance	Physical appearance, gestures, and mannerisms	“A well-appearing, clean young woman appearing her stated age, pleasant and cooperative. Eye contact was good."	Disheveled, clothing, groomed, wearing, clean
Thought Content	Suicidal/homicidal ideation, obsessions, phobias, delusions, hallucinations	“No SI, No HI, No hallucinations, Ideas of reference, Paranoid delusions"	Obsession, delusion, grandiose, ideation, suicidal, paranoid
Interpersonal	Family situation, friendships, and other social relationships	“Pt. overall appears to be functioning very well despite this conflict with a romantic interest of hers."	Boyfriend, relationship, peers, family, parents, social
Mood	Feelings and overall disposition	“Pt. indicates that his mood is becoming more ‘depressed.’"	Anxious, calm, depressed, labile, confused, cooperative
Occupation	School and/or employment	“Pt. followed through with decision to leave college at this point in time."	Boss, employed, job, school, class, homework, work
Thought Process	Pace and coherence of thoughts. Includes linear, goal-directed, perseverative, tangential, and flight of ideas	“Disorganized (Difficult to communicate with patient.), Paucity of thought, Thought-blocking."	Linear, tangential, prosody, blocking, goal-directed, perseverant
Substance	Drug and/or alcohol use	“Patient used marijuana once which he believes triggered the current episode."	Cocaine, marijuana, ETOH, addiction, narcotic
Other	Any example that does not fall into any of the other seven domains	“Maintain mood stabilization, prevent future episodes of mania, improve self-monitoring skills."	–

All sentences were removed from the surrounding EHR context to ensure annotators were not influenced by the additional contextual information. Our domain classification models consider each sentence independently and thus we designed the annotation task to mirror the information available to the models.

The annotators were instructed to label each sentence with one or more of the seven risk factor domains. In instances where more than one domain was applicable, annotators assigned the domains in order of prevalence within the sentence. An eighth label, ‘Other’, was included if a sentence did not align with any of the seven risk factor domains. The annotations were then reviewed by a team of two clinicians who adjudicated collaboratively to create a gold standard. Basic statistics on the corpus, including the number of sentences labeled for greater than one risk factor domain are listed in Table 3. The gold standard and the clinician-identified keywords and MWEs have received IRB approval for release to the community. They are available as supplementary data to this paper.

**Table 3:** Distribution of gold standard sentences and tokens across risk factor domains

	Total Sentences	Total Tokens
Appearance	670	11648
Mood	793	17672
Interpersonal	574	11674
Occupation	664	14166
Thought Content	756	18785
Thought Process	663	11203
Substance Use	727	14793
Totals	4847	99941
Sentences With >1 Domain	222	8912

#### Inter-Annotator agreement

Inter-annotator agreement (IAA) was assessed using a combination of Fleiss’s Kappa (a variant of Scott’s Pi that measures pairwise agreement for annotation tasks involving more than two annotators) [19] and Cohen’s Multi-Kappa as proposed by Davies and Fleiss [20]. Table 4 shows IAA calculations for both overall agreement and agreement on the first (most important) domain only. Following adjudication, accuracy scores were calculated for each annotator by evaluating their annotations against the gold standard.

**Table 4:** Inter-annotator agreement

Labels	Fleiss’s Kappa	Cohen’s Multi-Kappa	Mean Accuracy
Overall	0.575	0.571	0.746
First Domain Only	0.536	0.528	0.805

Overall agreement was generally good and aligned almost exactly with the IAA on the first domain only. Out of the 1654 annotated sentences, 671 (41%) had total agreement across all three annotators. We defined total agreement for the task as a set-theoretic complete intersection of domains for a sentence identified by all annotators.

98% of sentences in total agreement involved one domain. Only 35 sentences had total disagreement, which we defined as a set-theoretic null intersection between the three annotators. An analysis of the 35 sentences with total disagreement showed that nearly 30% included the term “blunted/restricted”. In clinical terminology, these terms can be used to refer to appearance, affect, mood, or emotion. Because the sentences being annotated were extracted from larger clinical narratives and examined independently of any surrounding context, it was difficult for the annotators to determine the most appropriate domain. This lack of contextual information resulted in each annotator using a different ‘default’ label: Appearance, Mood, and Other. During adjudication, Other was decided as the most appropriate label unless the sentence contained additional content that encompassed other domains, as it avoids making unnecessary assumptions.

A Fleiss’s Kappa of 0.575 lies on the boundary between ‘Moderate’ and ‘Substantial’ agreement as proposed by Landis and Koch [21]. This is a promising indication that our risk factor domains are adequately defined by our present guidelines and can be employed by clinicians involved in similar work at other institutions.

The fourth column in Table 4, Mean Accuracy, was calculated by averaging the three annotator accuracies as evaluated against the gold standard. This provides us with an informative baseline of human parity on the domain classification task.

### Topic extraction

**Fig. 1:**
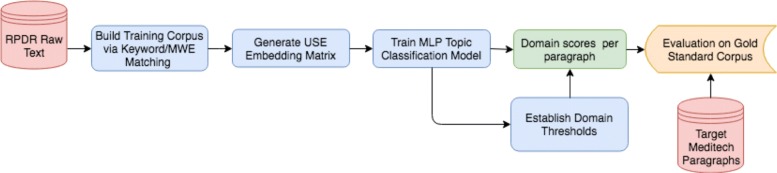
Data pipeline for training and evaluating our risk factor domain classifiers

Figure 1 illustrates the data pipeline for generating our training and testing corpora, and applying them to our classification models.

We use the Universal Sentence Encoder (USE) [22], a deep averaging neural network that is pretrained on a very large volume of general-domain web data, to convert sentences to 512-dimensional embedding vectors, stemming tokens with the Porter Stemmer tool provided by the NLTK library [23]. USE has the advantage of being sensitive to word ordering and can encode sequences of variable lengths, in addition to being integrated directly into TensorFlow. We have found in previous unpublished observations performed by Holderness, Meteer, Pustejovsky, and Hall that despite being pretrained on general-domain web data, USE outperforms other state-of-the-art embedding models such as ELMo, FastText, or Doc2Vec.

Starting with the approach taken by McCoy et al. [16], who used aggregate cosine similarity scores to compute domain similarity directly from a TF-IDF vector space model, we extend this method by training a suite of three-layer multilayer perceptron (MLP) and radial basis function (RBF) neural networks using a variety of parameters to compare performance. We employ the Keras deep learning library [24] using a TensorFlow backend [25] for this task. The architectures of our highest performing MLP and RBF models are summarized in Table 5. Prototype vectors for the nodes in the hidden layer of our RBF model are selected via *k*-means clustering [26] on each domain megadocument individually. The RBF transfer function for each hidden layer node is assigned the same width, which is based off the maximum Euclidean distance between the centroids that were computed using *k*-means.

**Table 5:** Architectures of our highest-performing MLP and RBF networks

Network	MLP	RBF
Input Layer		
Nodes	512	512
Dropout	0.2	0.2
Activation	ReLU	ReLU
Hidden Layer		
Nodes	250	700
Dropout	0.5	0.0
Activation	ReLU	RBF
Output Layer		
Nodes	7	7
Activation	Sigmoid	Linear
Optimizer	Adam	Adam
Loss Function	Categorical Cross Entropy	Mean Squared Error
Training Epochs	60	50
Batch Size	128	128

To prevent overfitting to the training data, we utilize a dropout rate [27] of 0.2 on the input layer of all models and 0.5 on the MLP hidden layer.

Since our classification problem is multiclass, multilabel, and open-world, we employ seven nodes with sigmoid activations in the output layer, one for each risk factor domain. This allows us to identify sentences that fall into more than one of the seven domains, as well as determine sentences that should be classified as Other. Unlike the traditionally used softmax activation function, which is ideal for single-label, closed-world classification tasks, sigmoid nodes output class likelihoods for each node independently without the normalization across all classes that occurs in softmax.

We find that the risk factor domains vary in the degree of homogeneity of language used, and as such certain domains produce higher similarity scores, on average, than others. To account for this, we calculate threshold similarity scores for each domain using the formula min=avg(sim)+ α* *σ*(sim), where *σ* is standard deviation and *α* is a constant, which we set to 0.5 for our P model and 1.25 for our RBF model through trial-and-error. Employing a generalized formula as opposed to manually identifying threshold similarity scores for each domain has the advantage of flexibility in regards to the target data, which may vary in average similarity scores depending on its similarity to the training data. If a sentence does not meet threshold on any domain, it is classified as Other.

## Results

Table 6 shows the performance of our MLP and RBF models on classifying the sentences in our gold standard. To assess relative performance of feature representations, we also include performance metrics of our models without MWEs. Because this is a multilabel classification task we compute precision, recall, and F1 scores for each sentence in the test set using macro-averaging, where performances are calculated for each risk factor domain individually and then averaged. In identifying the seven risk factor domains individually, our models achieved the highest per-domain scores on Substance (F1 ≈ 0.9) and the lowest score on Mood (F1 ≈ 0.75).

**Table 6:** Overall and domain-specific Precision, Recall, and F1 scores for our models

	Precision	Recall	F1
Aggregate Cosine Similarity Scores	0.626	0.692	0.657
MLP Baseline (No MWEs)	0.816	0.830	0.823
RBF Baseline (No MWEs)	0.795	0.808	0.801
MLP (w/ MWEs)	0.821	0.835	0.828
Appearance	0.953	0.825	0.884
Interpersonal	0.843	0.897	0.869
Mood	0.723	0.816	0.767
Occupation	0.945	0.834	0.886
Substance	0.898	0.946	0.921
Thought Content	0.830	0.685	0.751
Thought Process	0.792	0.878	0.833
Other	0.509	0.614	0.557
RBF (w/ MWEs)	0.814	0.799	0.806
Appearance	0.952	0.803	0.871
Interpersonal	0.929	0.882	0.905
Mood	0.748	0.759	0.754
Occupation	0.956	0.847	0.898
Substance	0.826	0.927	0.874
Thought Content	0.866	0.685	0.765
Thought Process	0.958	0.818	0.883
Other	0.405	0.411	0.408

**Fig. 2:**
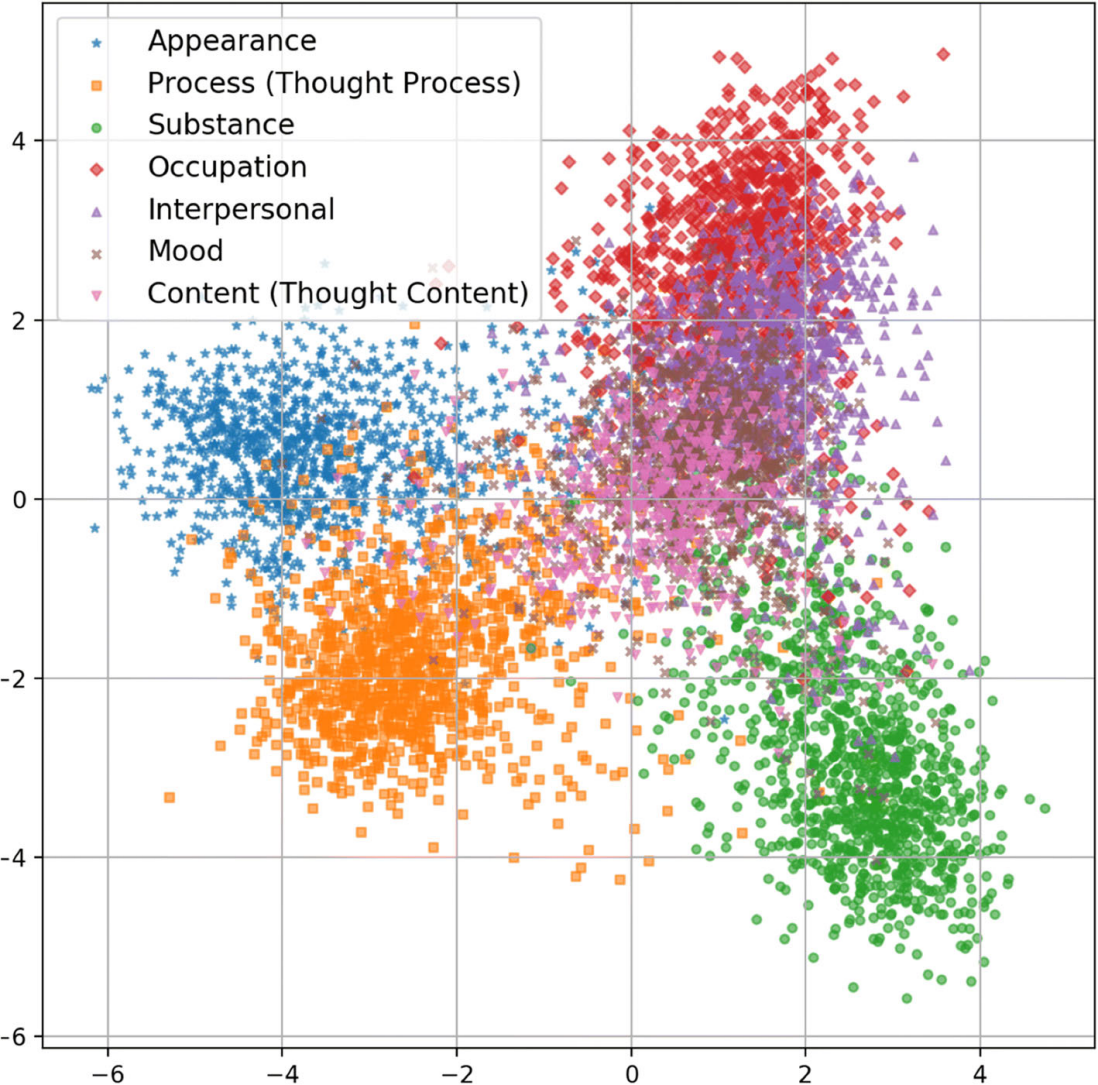
2-component linear discriminant analysis of the RPDR training data

Despite prior research indicating that similar classification tasks to ours are more effectively performed by RBF networks [28–30], we find that our MLP model performs marginally better with significantly less computational complexity (i.e. *k*-means and width calculations). Figure 2 illustrates the distribution of sentences in vector space using 2-component Linear Discriminant Analysis (LDA) [31], and shows that Thought Process, Appearance, Substance, and – to a certain extent – Occupation clearly occupy specific regions, whereas Interpersonal, Mood, and Thought Content occupy the same noisy region where multiple domains overlap. In RBF networks, the magnitude of activation for a given hidden layer neuron is based on the Euclidean distance from the input vector to the prototype centroid associated with that neuron. Smaller distances lead to more robust activations. To identify these prototype centroids, we apply the *k*-Means clustering algorithm to identify the training examples for each class that most closely describe the distribution of the examples in vector space. With large training sets such as ours, the RBF prototype centroids will be more precise and therefore the RBF model is more powerful in differentiating between classes in crowded regions of vector space. This is reflected by the results in Table 6, where the RBF network performs as well as or stronger than the MLP network in the four overlapping domains (0.905 vs 0.869 for Interpersonal, 0.754 vs 0.767 for Mood, 0.898 vs 0.886 for Occupation, and 0.765 vs 0.751 for Thought Content) whereas the MLP network – with the exception of Thought Process – performs as well as or stronger than the RBF network when predicting the non-overlapping domains (0.874 vs 0.921 for Substance, 0.871 vs 0.884 for Appearance, and 0.883 vs 0.833 for Thought Process). We also observe a similarity in the words and phrases with the highest Term Frequency – TF-IDF scores across the overlapping domains: many of the Thought Content words and phrases with the highest TF-IDF scores involve interpersonal relations (e.g. ‘fear surrounding daughter’, ‘father’, ‘family history’, ‘familial conflict’) and there is a high degree of similarity between high-scoring words for Mood (e.g. ‘meets anxiety criteria’, ‘cope with mania’, ‘ocd’) and Thought Content (e.g. ‘mania’, ‘feels anxious’, ‘feels exhausted’). Please refer to the “Methods” section of this paper for more information on our TF-IDF analysis and its implications in building our training corpus.

The most significant discrepancy in model performances is in classifying sentences that do not involve any of the seven risk factor domains. While both are fairly inaccurate at identifying these ‘Other’ sentences, our MLP model has a markedly higher F-score (0.557) compared to our RBF model (0.408).

## Discussion

Results clearly indicate that our MLP and RBF deep learning models outperform the cosine similarity baseline. Additionally, our models are more scalable and computationally efficient to handle large volumes of data. In our initial work on risk factor domain topic extraction with a training data set of only 100,000 sentences, we found performance to increase by 15% when factoring in MWEs, a marked improvement over our models that did not incorporate them. However, with our current training data set of 2,100,000 sentences, factoring MWEs into our models increased classification performance by only 1% uniformly across all risk factor domains, both overlapping and non-overlapping. This aligns with our expectations that MWEs comprised of a quotidian vocabulary hold more clinical significance than when the words in the expressions are treated independently but that as the amount of training data increases, these MWEs are captured organically. Even with the larger volume of training data, the clinician-identified keywords and MWEs continue to play an important role when generating the training data set, as training sentences are identified by regular expression pattern matching of these keywords and MWEs. Therefore, including MWEs at this step increases the scope and variety of training sentences, leading to more robust downstream performance.

The wide variance in per-domain performance is due to a number of factors. Most notably, the training sentences we extracted from RPDR – while very comparable in structure and style to our target OnTrack^TM^ data – may not have an adequate variety of content and range of vocabulary. Although using keyword and MWE matching to create our training corpus has the advantage of being significantly less labor intensive than manually labeling every sentence in the corpus, it is likely that the homogeneity of language used in the training sentences is higher than it would be otherwise. Additionally, all of the sentences in the training data are assigned exactly one risk factor domain even if they actually involve multiple risk factor domains, making the clustering behavior of the sentences more difficult to define.

Threshold similarity scores also play a large role in determining the precision and recall of our models: setting higher classification thresholds leads to a smaller number of false positives and a greater number of false negatives for each risk factor domain. Conversely, more sentences are incorrectly associated with one or more risk factor domains when thresholds are set lower. Since our classifier will be used in future work as an early step in a data analysis pipeline for determining readmission risk, misclassifying a sentence with an incorrect risk factor domain at this stage can lead to greater inaccuracies at later stages. Sentences misclassified as Other, however, will be discarded from the data pipeline. Therefore, we intentionally set a conservative threshold where only the most confidently labeled sentences are assigned membership in a particular domain. In addition to the challenges associated with fine-tuning threshold similarity scores, Other as a domain is much broader in scope than the seven risk factor domains, encompassing most of the space surrounding the clusters in Fig. 2. Because the function describing this space is more complex than the functions delineating the regions of vector space occupied by the specific risk factor domains, model accuracy is predictably lower when classifying these out-of-domain examples.

The IAA that we report on our annotation task falls in the upper end of ’Moderate’ agreement and is only 0.03 away from being considered ’Substantial’ agreement as proposed by Landis and Koch [21]. From a clinical psychiatric perspective, it is in fact satisfactory and the first of its kind in the psychosis clinical NLP literature. As described in the Background Section, dealing with clinical narratives in psychotic EHRs are challenging for a number of reasons. Also, our annotation task is multiclass, multilabel, and open-world (i.e., 7 risk factor domains plus “other” for sentences that are not relevant to those domains), making high IAA very difficult to achieve. The degree of difficulties specific to each domain also affect the overall IAA. Some domains such as “Substance” produced high IAA because it is easy for annotators to agree on sentences involving substance (e.g., cocaine, cannabis). Whereas other domains with a larger vocabulary overlap, such as Mood, Thought Content, and Interpersonal are more challenging (see Fig. 2). For example, “Pt is a 32 year old single Caucasian male with a history of Schizoaffective Disorder, two prior psychiatric hospitalizations, with increasing disorganized thought process, paranoia, and command auditory hallucinations in the context of discontinuing his psychiatric medications” was annotated to be “Thought Process” & “Thought Content” by two annotators and “Appearance” & “Thought Content” & “Thought Process” by the third annotator, resulting in partial agreement among annotators. For each sentence, the gold standard was created by a majority agreement among annotators when two or more annotators were in total agreement. For the remaining sentences, high quality, domain-expert adjudications were made by a team of two clinicians who worked collaboratively. Therefore, we believe that the resulting corpus can be used as a “gold standard”.

In terms of computational complexity, our MLP model significantly outperforms our RBF model during training and outperforms both the RBF model and the cosine similarity baseline at evaluation. Whereas our MLP model trains in O(n) time, requiring only one pass through each datapoint for each epoch of training, our RBF model trains in O(n^w^) time, where w is equivalent to the number of prototype centroids in the hidden layer, as each training example is evaluated against each prototype centroid in the network. In addition, the *k*-Means clustering that must be performed before training the RBF network to identify the prototype centroids runs in O(n) time. Although the cosine similarity baseline model does not have a training phase, it runs in O(n^2^) at evaluation since the distance between each element in the test corpus and each element in the training corpus must be computed.

Although both the RBP and MLP models performed roughly equivalently, the MLP is a simpler model and is faster to train and evaluate compared to an RBF network. Given the intention of implementing this model in a larger clinical NLP pipeline, the lower latency MLP model is preferred.

## Conclusions

To achieve our goal of creating a framework for a readmission risk classifier, the present study performed necessary evaluation steps by updating and adding to our model iteratively. In the first stage of the project, we focused on collecting the data necessary for training and testing, and on the domain classification annotation task. At the same time, we began creating the tools necessary for automatically extracting domain relevance scores at the sentence and document level from patient EHRs using several forms of vectorization and topic modeling. In future versions of our risk factor domain classification model we will explore increasing robustness through sequence modeling that considers more contextual information.

Our current feature set for training a machine learning classifier is relatively small, consisting of sentence domain scores, bag-of-words, length of stay, and number of previous admissions, but we intend to factor in many additional features that extend beyond the scope of the present study. These include a deeper analysis of clinical narratives in EHRs: in a different line of development, we have extended our EHR data pipeline by distinguishing between clinically positive and negative phenomena within each risk factor domain [32]. This involved a series of annotation tasks that allowed us to generate lexicon-based and corpus-based sentiment analysis tools. In future work, we intend to use these clinical sentiment scores to generate gradients of patient improvement or deterioration over time with respect to each of the seven risk factor domains for readmission.

We will also take into account structured data that have been collected on the target cohort throughout the course of this study such as brain based electrophysiological (EEG) biomarkers, structural brain anatomy from MRI scans, social and role functioning assessments, personality assessments (NEO-FFI), and various symptom scales (PANSS, MADRS, YMRS). For each feature we consider adding, we will evaluate the performance of the classifier with and without the feature to determine its contribution as a predictor of readmission.

## Data Availability

All data generated or analyzed during this study are included in this published article and its Additional files.

## Abbreviations

Adam: Adaptive Moment Estimation [33]
EHR: Electronic Health Record
ETOH: Ethyl alcohol and ethanol
HI: Homicidal ideation
IAA: Inter-Annotator Agreement
MADRS: Montgomery-Asperg Depression Rating Scale [34]
MLP: Multi-Layer Perceptron
MWE: Multi Word Expression
NEO-FFI: NEO Five-Factor Inventory [35]
NLTK: Natural Language Toolkit
OCD: Obsessive-compulsive disorder
PANSS: Positive and Negative Syndrome Scale [36]
RBF: Radial Basis Function
ReLU: Rectified Linear Units, *f*(*x*)=*max*(0,*x*) [37]
RPDR: Research Patient Data Registry
SI: Suicidal ideation
TF-IDF: Term Frequency – Inverse Document Frequency
USE: Universal Sentence Encoder YMRS: Young Mania Rating Scale [38]

## Footnotes

1 This study has received IRB approval.

2 The vast majority of patients in our target cohort are dependents on a parental private health insurance plan.
